# Electrocardiographic Reference Values in Clinically Healthy Lusitano Horses

**DOI:** 10.3390/vetsci10080518

**Published:** 2023-08-10

**Authors:** Alexandre Triguinho, Ana Patrícia Fontes-Sousa, José Pimenta, Mário Cotovio

**Affiliations:** 1 Independent Veterinary Care, Chapelfield Veterinary Partnership, Brooke Equine Clinic, Bungay Road, Brooke, Norwich NR15 1DX, UK; 2 Department of Immuno-Physiology and Pharmacology, Abel Salazar Institute of Biomedical Sciences, University of Porto (ICBAS-UP), 4050-313 Porto, Portugal; 3 Center for Drug Discovery and Innovative Medicines (MedInUP), Abel Salazar Institute of Biomedical Sciences, University of Porto (ICBAS-UP), 4050-313 Porto, Portugal; 4 UPVET, Veterinary Hospital of Abel Salazar Institute of Biomedical Sciences, University of Porto (ICBAS-UP), 4050-313 Porto, Portugal; 5 Department of Veterinary Sciences, University of Trás-os-Montes e Alto Douro (UTAD), 5001-801 Vila Real, Portugal; 6 CECAV—Veterinary and Animal Research Center, University of Trás-os-Montes e Alto Douro, 5000-801 Vila Real, Portugal; 7 Associate Laboratory for Animal and Veterinary Sciences (AL4AnimalS), 5000-801 Vila Real, Portugal

**Keywords:** electrocardiogram, reference values, Lusitano horse

## Abstract

**Simple Summary:**

The electrocardiogram is a relevant diagnostic tool for detecting cardiac rhythm disturbances during routine examinations. With the increasing importance of horses as sport animals, cardiology now plays a significant role in equine medicine. In particular, the Lusitano horse has gained worldwide acceptance for its athletic potential. However, the breed’s unique characteristics can influence electrocardiogram results, making it essential to establish reference values for accurate evaluations. Currently, there are no established reference values for the Lusitano horse, and this study aims to bridge this gap. This study involved electrocardiographic evaluations of 82 clinically healthy adult Lusitano horses using lead II and the base–apex lead. The results revealed a median heart rate of 39 beats per minute. The P wave exhibited mainly a bifid configuration, while the QRS complex appeared in several forms; QR and R were the most frequent configurations in lead II, while RS was the most common in the base–apex lead. Most T waves had a biphasic shape in both methods. Statistically significant differences were observed among genders and ages. These results underscore the importance of establishing reference values for athletic horses to improve the accuracy of cardiac evaluations.

**Abstract:**

The Lusitano horse is gaining popularity in the equestrian world, and as a result, the significance of applied sports medicine for this breed is growing. As cardiology plays a crucial role in this field, numerous studies have been conducted to establish electrocardiographic reference values in various breeds to ensure a more accurate evaluation. However, studies regarding healthy Lusitano horses are lacking. So, this study aimed to establish electrocardiographic reference values for Lusitano horses, utilizing a sample of 82 clinically healthy animals. The evaluation involved lead II and base–apex lead measurements, with a median heart rate of 39 beats per minute being recorded. The P wave demonstrated a predominantly bifid configuration, while the QRS complex exhibited various forms. The most common QRS configurations were QR and R in lead II, and RS in the base–apex lead. Additionally, most T waves displayed a biphasic shape in both methods. Furthermore, statistically significant differences were noted based on age and gender. Some of the electrocardiographic values obtained differed from those previously published for other breeds. Given the relevance of electrocardiogram in cardiovascular evaluation, these findings bring valuable insights regarding the specific parameters for Lusitano horse and emphasize the importance of obtaining breed-specific electrocardiographic reference values.

## 1. Introduction

The electrocardiogram (ECG) is a well-established diagnostic tool used for a comprehensive cardiac evaluation in humans and veterinary medicine, including horses. Electrocardiography is the most reliable method for detecting cardiac dysrhythmias and conduction disturbances, which are relatively common in horses [1,2,3,4]. Additionally, this complementary exam enables the clinician to differentiate between physiological arrhythmias and pathological ones caused by valvular, myocardial, or systemic diseases [5,6,7,8,9]. Physiological arrhythmias are typically linked to the naturally high vagal tone in horses, which may cause non-pathologic variations of the normal sinus rhythm, such as second-degree atrioventricular or sinus blocks. These variations should disappear with exercise [3]. It is crucial to have high-quality ECG recordings to distinguish between normal and pathological complexes and artifacts. With the abundance of medical technologies available today, equine practitioners are increasingly able to record an ECG in a horse at rest, for extended periods of time, or during exercise. With numerous portable or easily connected ECG recording devices that can communicate digitalized data wirelessly, recordings are quickly obtained in a field situation. This also makes it possible for quick data sharing amongst colleagues, which occurs often during prepurchase exams, to discuss the possible implications of alterations found during cardiovascular/ECG examination [2]. 

The Lusitano horse is considered one of the most ancient equine breeds in the world. It originates from Portugal, where it is economically the most important horse breed, with a registered population of about 5000 mares and 1000 stallions [10,11]. The breed has expanded worldwide and is reared in many countries, with Brazil recording the second largest population, followed by France and then Spain [10,11]. Over time, the Lusitano horse has demonstrated a great aptitude for carriage driving, working equitation, and mostly dressage, with an increasing number of participants in recent years, both at national and international competitions [12]. This international projection allowed the Lusitano horse to participate in equestrian events organized by the International Equestrian Federation (FEI). The FEI, together with the World Breeding Federation for Sport Horses (WBFSH), is responsible for maintaining and conducting the international rankings of horses, riders, and breeders’ associations for each of the equestrian disciplines they organize, publishing these rankings at the end of every competitive season [13]. Within the associations evaluated, the Breeders Association of Lusitano Horses (APSL) has been ranked among the top ten [13,14].

Therefore, the significance of applied sports medicine for this breed is increasing, with cardiology playing a crucial role in achieving optimal performance [15]. While ECG have been extensively studied in various species, the importance of studying the difference of ECG parameters between breeds cannot be overstated. Horses exhibit significant variations in their physiological characteristics, including body and heart size, conformation, and inherited athletic capabilities, which may influence their ECG evaluation [16]. By recognizing the impact of these breed-specific differences on electrocardiographic examination, veterinarians and researchers can improve the accuracy of cardiac examination and diagnosis. Furthermore, generalized reference values might not sufficiently account for the unique characteristics of individual horses belonging to specific breeds. This emphasizes the importance of establishing breed-specific reference values to facilitate a more accurate interpretation of ECG results, minimize the potential for misdiagnosis, and ensure appropriate clinical follow-up for horses. Several studies have been conducted to establish ECG reference values for different horse breeds, indicating that breed-specific factors can impact these measurements [17,18,19,20,21,22]. However, to the best of the authors’ knowledge, there is currently no available information on ECG parameters for clinically healthy Lusitano horses. In this context, we conducted a study on Lusitano horses to establish breed-specific electrocardiographic reference values. We utilized the bipolar II limb and the base–apex lead systems and compared our findings with existing studies on other breeds. Furthermore, we investigated the potential influence of gender, age, and weight on ECG measurements in Lusitano horses.

## 2. Materials and Methods

### 2.1. Animal Selection Criteria 

This study was carried out on 82 clinically healthy Lusitano horses, sourced from diverse owners and regions across Portugal. All owners gave their informed consent for the use of their animals’ data. The study was ethically approved by the Animal Welfare Organism of the University of Trás-os-Montes e Alto Douro (UTAD)-Portugal (ORBEA) as complying with the Portuguese legislation for the protection of animals (Law no. 6 January 2022). For the present study, inclusion and exclusion criteria were established prior to ECG. Only healthy animals, which had received routine deworming and vaccination against tetanus and influenza in the previous 6 months, were included. The owners or caretakers of each horse confirmed their normal physical condition, regular activity, and absence of disease or health problems in the previous 6 months. Before performing ECG, a complete physical examination was performed on all animals, which included visual inspection, rectal temperature measurement, assessment of heart and respiratory rates, examination of mucous membranes and capillary refill time, as well as auscultation of the lungs, heart, and abdomen. Unhealthy animals, those that had undergone surgery or received any medication in the previous 6 months, as well as pregnant and lactating mares, were excluded from the study.

Information regarding gender and age for each animal was collected. The weight measurement method followed the procedure described by [23]. In brief, the body length was measured from the point of the shoulder to the ischial tuberosity, and the heart girth circumference was measured using a plastic measuring tape placed behind the elbow, passing in a straight vertical line over the withers and across the sternum. These measures were then used in the following formula: estimated weight (Kg) = (heart girth^2^ × body length)/(11.880 cm^3^). For statistical analyses, the horses were categorized into three age groups as mentioned in [24,25]: Group 1 (3–5 years old), Group 2 (6–14 years old), and Group 3 (>14 years old).

### 2.2. Electrocardiogram Procedure

We obtained three electrocardiograms (ECGs) from each horse using a three-channel Auto Cardiner FCP-145u Fukuda Denshi electrocardiograph. The ECGs were recorded using the bipolar II limb and the base–apex lead systems, with a paper speed of 25 mm/s and a sensitivity of 10 mm/mV. Electrocardiograms were performed in the horses’ habitual facilities, avoiding transport or changes in the environment of the animals. The horses were standing in a familiar location outside their stall and were allowed to acclimate for at least 15 min before conducting the exams.

Prior to applying alcohol, we attached alligator clips fixed to the electrocardiographic electrodes directly to the skin. Clipping was not necessary since the air coat of the horses was not too long, which allowed those electrodes to stay in tight contact with the horse skin. For bipolar II limb lead recordings, electrodes were positioned fifteen centimeters below the level of the olecranon on the caudal aspect of the forelimbs and distal to the stifle joint on the hind limbs, as previously described [1,26]. During the exam, the forelimbs were kept parallel to each other and perpendicular to the long axis of the body as much as possible. The negative electrodes were placed on the forelimbs, and the positive and neutral electrodes on the left and right hind limbs, respectively. 

**Figure 1:**
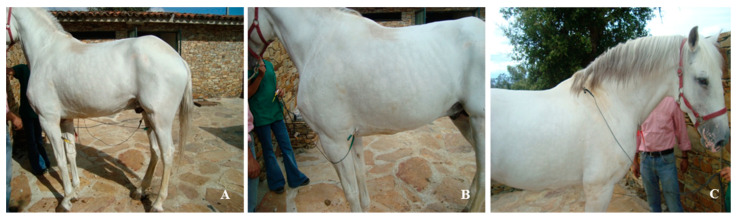
Electrodes placement for bipolar II limb lead (**A**,**B**) and for base–apex lead (**C**): (**A**) electrodes distal to the stifle joint on the hind limbs; (**B**) electrodes below the level of elbow; (**C**) electrodes in the lower third of the right jugular groove and in the right hemithorax, dorsal to the scapula.

For base–apex lead recordings, the positive electrode was placed on the left thorax in the fifth intercostal space at the level of the elbow. The negative electrode was positioned in the lower third of the right jugular groove, and the neutral electrode on the right hemithorax, dorsal to the scapula. After placing the electrodes, a second period of acclimation was allowed, for the horses to adapt to the alligator clips. The positions of the electrodes are illustrated in Figure 1.

### 2.3. Electrocardiogram Evaluation

The recordings were first analyzed in their entirety to identify any arrhythmias, and then digitized for further analysis. Three consecutive, regular, and artefact-free cardiac cycles from each lead were selected to evaluate heart rhythm and to examine the configuration, amplitude, and duration of the waves (including the P wave, QRS complex, and T wave), as well as the duration of the PR and QT intervals [17,22,26]. To measure RR intervals, we identified the area of the recording where the heart rate was most consistent. If a sinus or atrioventricular block was present, the corresponding heart cycle was excluded from our analysis of electrocardiographic variables. If a significant variation in any of the measured variables was detected between the three selected cardiac cycles, we conducted one to two additional measurements to improve the reliability of results.

### 2.4. Statistical Analysis

Statistical analysis was performed using the SPSS program, version 19. The Kolmogorov–Smirnov test was used to assess whether the continuous variables followed the normal distribution. Descriptive statistics were obtained for clinical information and electrocardiographic measurements, being presented in the form of median (interquartile range—IQR). The Mann–Whitney test and Kruskal–Wallis test were used to examine differences in ECG variables between genders and age categories, respectively. The Spearman correlation test was used to evaluate the correlation of age, weight, and gender in ECG parameters. The level of significance was defined as *p* < 0.05. 

The calculation of reference intervals and their confidence interval limits (90%) was carried out using Reference Value Advisor V 2.1 software, a free set of macroinstructions designed to calculate reference intervals with Microsoft Excel [27].

## 3. Results

### 3.1. Animals’ Data

A total of 82 animals were analyzed, consisting of 19 females and 63 males. The animals’ ages ranged from 3 to 22 years old, with a median age of 8 (7) years. Their median body weight was 450 (50) kg. The median heart rate was 39 (12) beats per minute (bpm). 

### 3.2. Electrocardiogram Reference Values

The ECG reference values for the base–apex lead are presented in Table 1, while the reference values for lead II are provided in Table 2. For certain ECG parameters, reference values could not be determined in both leads due to small sample sizes in those specific parameters. Amplitude is presented in millivolts (mV) and duration in seconds (s).

### 3.3. Electrocardiogram Evaluation 

The sinus rhythm was the predominant heart rhythm in all the evaluated horses. However, a subset of horses (14/82; 17.5%) exhibited intermittent arrhythmias, including sinus block (3/82) and second-degree atrioventricular blocks (4/82), with one horse exhibiting both sinus block and second-degree atrioventricular block. Sinus bradycardia (1/82), sinus tachycardia (2/82), and sinus arrhythmia (4/82) were also detected.

**Figure 2:**
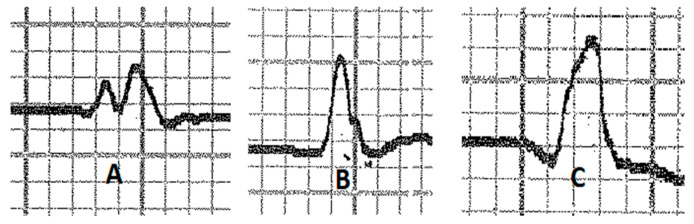
P wave configurations: (**A**) bifid; (**B**) simple; (**C**) biphasic.

**Figure 3:**
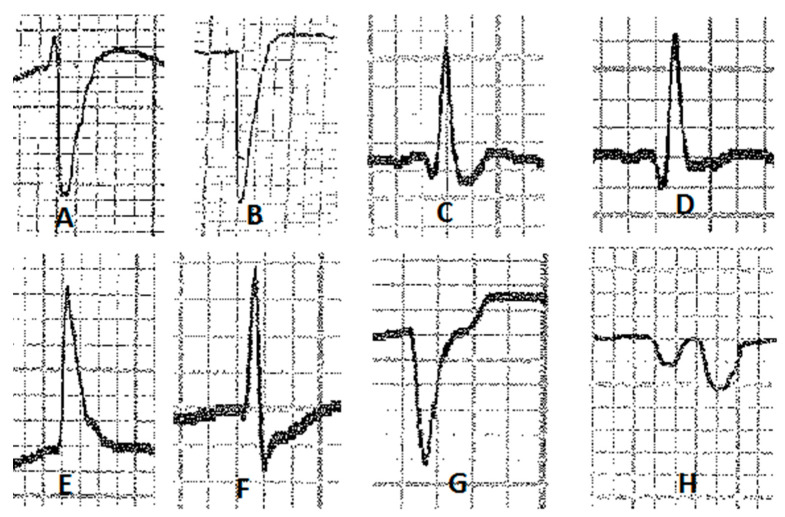
QRS complex configurations: (**A**) RS complex (base–apex)*;* (**B**) S wave (base–apex)*;* (**C**) QRS complex (lead II); (**D**) QR complex (lead II); (**E**) R wave (lead II); (**F**) RS complex (lead II); (**G**) S wave (lead II); (**H**) QS complex (lead II).

**Figure 4:**
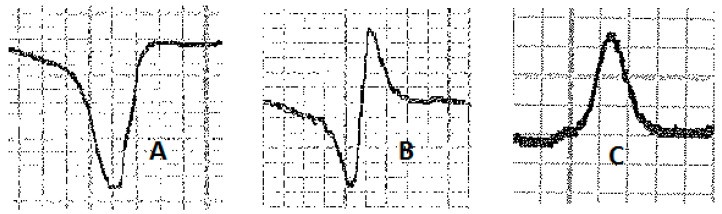
T wave configurations: (**A**) simple negative T wave; (**B**) biphasic T (+/−) wave; (**C**) simple positive T wave.

Various configurations of P waves were observed (Figure 2). In lead II, we observed a bifid P wave (P1 and P2) in 63.4% of the recordings, while a simple P wave (P+) was present in 36.6% of cases. No biphasic (P− and P+) waves were recorded. In the base–apex lead, 87.8% of recordings showed a bifid P wave, while 9.8% showed a simple P wave and 2.4% showed a biphasic P wave. We observed six different types of QRS complexes (Figure 3) in lead II, with QR (25.6%), R (23.2%), and QRS (22%) configurations being more common than RS (13.4%), S (11.8%), and QS (4%). In the base–apex lead, three configurations of QRS complexes were found: RS (62.2%), S (35.4%), and QRS (2.4%). Additionally, the T wave in lead II and the base–apex lead showed three configurations (Figure 4), with the biphasic (T− and T+) pattern being the most common in both leads (73.2% and 82.9%, respectively). The simple negative and simple positive configurations of the T wave were found in 12.2% and 13.4% of horses, respectively, in lead II, while in the base–apex lead, they were found in 10.5% and 7.3% of horses, respectively.

### 3.4. Comparison of Groups 

The median amplitude of the P2 and R waves in lead II was found to be lower in females compared to males, with significant statistical differences (*p* = 0.012 and *p* = 0.001, respectively). Furthermore, in the base–apex lead, females did not exhibit a simple P wave configuration or a Q wave in the QRS complex. The females exhibited a higher median heart rate compared to males (40 bpm vs. 36 bpm) with significant statistical difference (*p* < 0.05). This difference was reflected in a longer RR interval for males in both leads (*p* < 0.05). Table 3 shows the results obtained from the various electrocardiographic measurements by gender.

When analyzing different age groups, lead II recordings revealed that the RR interval was longer in Group 1 (3–5 years; *p* < 0.05) compared to Group 3 (>14 years), while the QT interval was longer in Group 3 (*p* < 0.01) than in Groups 1 and 2. In the base–apex lead, animals from Group 3 exhibited longer QRS complexes and T waves than Group 1 (*p* < 0.01 for QRS complex; *p* < 0.05 for T wave) and Group 2 (*p* < 0.05 for QRS complex; *p* < 0.01 for T wave). Table 4 shows the results obtained from the various electrocardiographic measurements by age.

In lead II, a positive correlation was observed between age and the duration of the QRS complex (*p* = 0.012), T wave (*p* = 0.015), and QT interval (*p* = 0.007). Additionally, in the base–apex lead, age exhibited a positive correlation with the duration of the P wave (*p* = 0.020) and the duration of the QRS complex (*p* = 0.007). However, no correlations were found between weight and any of the electrocardiographic characteristics. Furthermore, gender was found to be correlated with certain ECG parameters in both lead II and the base–apex lead. In lead II, males presented a longer duration of the P wave (*p* = 0.012), as well as greater amplitudes of the P2 wave (*p* = 0.004) and R wave (*p* = 0.001). In the base–apex lead, males showed a shorter duration of the P wave (*p* = 0.027) and a longer amplitude of the P2 wave (*p* = 0.004).

## 4. Discussion

Cardiovascular alterations are the third most common cause of poor performance in horses, following musculoskeletal and respiratory diseases. The cardiovascular system plays a crucial role in equine performance by maintaining normal thermoregulation and controlling blood flow to vital organs. However, the clinical evaluation of equine cardiovascular system is often challenging and complex. Horses are known to exhibit a range of physiological arrhythmias (e.g., sinus pause, sinus arrhythmia, first- and second-degree atrioventricular blocks) and murmurs. Therefore, during clinical assessment, it is important to be aware of the normal variations within the species [3,4,16,28]. Accurate assessment of the cardiovascular state relies on a thorough clinical examination and the ability to choose and interpret different complementary diagnostic tests such as ECG. Incomplete information may result in failure to detect subtle variations of normality that can impact equine performance. There have been significant advancements in cardiology research in recent decades, particularly in the field of complementary diagnostic modalities [3,4,16,28].

Different breeds have different physical characteristics such as weight and size that create differences in ECG parameters. For example, small equine breeds tend to have slightly higher heart rates and shorter ECG time intervals, such as the PR interval, QRS interval, and QT interval, compared to larger equine breeds [3,4]. The Lusitano horse is an extremely versatile sport horse and is the most important native equine breed in Portugal. Investigation of the physiological features of this breed is imperative to ensure a rational use of Lusitano sport horses and to improve purebred Lusitano horses’ potential through selection and breeding programs [10,11,12,29]. Several publications have reported electrocardiographic reference values for various breeds of horses [17,18,19,20,21,30]. However, to the best of our knowledge, this is the first study describing in detail the electrocardiographic reference values in resting Lusitano horses. In this discussion, we compare our results only with studies where the electrodes’ placement was similar to ours since electrode placement can affect ECG parameters, as previously reported [31].

The lack of appropriate breed-specific RIs for ECG parameters leads clinicians to use generic RIs present in the literature. Research about this topic can highlight the main differences between breeds and can help to clarify whether the use of generic reference values may compromise the interpretation of ECG results. The comparison of RIs between studies is difficult to perform due to the use of different ECG methodologies which can influence the results. Additionally, of the few existing studies in this area, many of them do not present true reference values, but rather present mean values and the standard deviation of the electrocardiographic parameters. Costa et al. 1985 [32] presented RIs for some ECG parameters in non-trained horses and some slight differences can be observed. The T wave duration in those horses presented an RI of 0.06–0.20 s, lower than the Lusitano horses of our study which presented 0.08–0.31 s. In contrast, the QT interval RI was 0.35–0.59 s and in our study the RI was 0.41–0.63 s. The other ECG parameters did not present significant differences, namely, the QRS complex duration (0.04–0.14 s) and P wave duration (0.06–0.16 s), compared with our study that presented 0.04–0.16 s and 0.06–0.15 s, respectively. Tavanaei-Manesh H et al. 2010 also presented some differences in the RIs measured in Purebred Kurd horses, namely, in QRS complex duration (0.10–0.16 s), T wave duration (0.12–0.20 s), and QT interval duration (0.44–0.64 s), when compared to the Lusitano horses of the present study (QRS complex duration: 0.04–0.16 s, T wave duration: 0.08–0.31 s, and QT interval duration: 0.41–0.63 s). Schwarzwald 2018 also presented some modest differences in RIs, namely, in QT interval duration (0.36–0.60 s) and QRS complex duration (0.08–0.14 s), compared with our work that presented 0.41–0.60 s and 0.04–0.16 s, respectively.

Melchert et al. 2012 compared certain electrocardiographic values from 23 Lusitano horses before and after exercise. In comparison with our study, no significant differences were found regarding arrhythmias. For sinus arrhythmia, we observed it in 4.87% of the horses, whereas Melchert et al. 2012 reported a prevalence of 4.16% in their study. Second-degree atrioventricular block was observed in 4.87% of the horses in our study, and Melchert et al. 2012 mentioned it in 4.16% of the horses. The differences were more pronounced when considering sinus tachycardia, as we observed a prevalence of 2.43%, while the aforementioned authors reported a prevalence of 4.16% [33].

In the base–apex lead, the ECG traces showed three different configurations for P waves: bifid, simple positive, or biphasic. Consistent with previous studies [18,20,34], the bifid and biphasic configurations were the most (88.2%) and least (2.6%) observed, respectively [4,9,18,34]. The duration and the amplitude of the P waves observed in this study were similar to those reported for Andalusian [35] and Mangalarga Marchador [20] breeds, which are genetically and morphologically close or related to Lusitano horses [36].

Our study revealed that in lead II, most P waves exhibited a bifid configuration (67.6%), consistent with previous findings in Andalusian horses (45%) [19]. Interestingly, the percentage of bifid P waves was even higher in the base–apex lead (88.2%), which could be attributed to the horses being more tolerant and calmer during electrode placement in this lead. Notably, the P1 component of the P wave is strongly influenced by the autonomic nervous system and is more prominent when vagal tone increases [21]. 

The configuration of the QRS complex depends on the different pathways taken by the cardiac impulse during ventricular depolarization [37]. In horses, the QRS complex assumes a very labile configuration, with the amplitude of each wave also being highly variable [38]. This variability can be attributed mainly to the nature of the Purkinje system in the equine species. In horses, the Purkinje fibers are widely distributed through the myocardium, and penetrate the entire thickness of the ventricular walls [16]. This leads to significant variations in the pathways of ventricular activation and the duration of the QRS complex itself. Additionally, it results in a “burst” type of depolarization of the major masses of both ventricles and the middle portion of the septum. During this type of depolarization, the electrical impulse does not follow any specific direction and therefore contributes poorly to the formation of the QRS complex [39].

Three different QRS complex configurations were observed in the Lusitano horse when analyzing the base–apex lead. The most common configuration (61.9%) was RS, consistent with other breeds [18,20,34]. However, the remaining configurations detected (S, 36.8%, and QRS, 1.3%) differed from those described in Purebred Kurd [18] and Standardbred horses [37] but were similar to those of Mangalarga Marchador horses [20].

In lead II, the QRS complex also exhibits several configurations [4]. However, the literature lacks information on the prevalence of each configuration and only reports the percentage of occurrence of each wave individually. If we adopt this approach, the Q wave was present in 62%, the R wave in 85.3%, and the S wave in 50.7% of the animals. These results are like those found in Thoroughbreds and Standardbreds, where R and S waves were the most and least common, respectively [21]. However, the R wave was present in 100% of horses in both breeds, and the Q wave was recorded in 90% of Thoroughbreds and 97.5% of Standardbreds. This difference could be attributed to distinct patterns of distribution of the Purkinje fibers and, consequently, different pathways followed by the cardiac impulse during ventricular activation [39].

The amplitudes of QRS waves obtained in this study diverged from those reported in the literature. Compared to Lusitano horses, Thoroughbred and Standardbred horses tended to exhibit lower amplitudes of negative waves (Q and S) and higher amplitudes of the R wave in lead II. 

There are several factors, such as excitation, stress, and exercise, that can disrupt T wave patterns [26]. The T wave patterns observed in the base–apex lead were consistent with those found in other breeds [18,34,40]. Negative T waves, whether monophasic (10.5%) or biphasic (82.9%), are considered a reliable indicator of myocardial oxygenation and equine health [34]. The duration of the T wave was consistent with the findings of several studies in other breeds like Purebred Kurd, Turkman horse, and Italian Trotter horses [18,34,40]. In lead II, the distribution of T wave configurations (biphasic, 76%; simple negative, 12%; simple positive, 12%) was consistent with the literature [19,37]. However, in adult Andalusian horses, the percentage of negative T waves, either as simple negative or as part of a biphasic configuration, was lower than in our study (61% vs. 88%). The amplitude of the T wave was in accordance with previous studies, but its duration was longer than what was found in the literature [30,32,40].

In the base–apex lead, it has been hypothesized that PR and QT intervals are comparable among breeds, and the current study supported this theory [9,18,20,34]. In lead II, the PR interval differed from other breeds, which may have been due to horses having less tolerance for electrode placement in the limbs than in the base–apex lead, as mentioned earlier. 

Gender demonstrated a positive correlation with electrocardiographic variables. Males exhibited a lower heart rate, resulting in a longer RR interval, consistent with previous studies [18]. In lead II, males showed larger P2 and R wave amplitude than females. However, it is important to note that the female group was relatively small, so these results should be interpreted with caution. Concerning the age, the present study observed a positive correlation with the electrocardiographic characteristics evaluated, namely, in the base–apex lead, older Lusitano horses (>14 years) exhibited a longer duration of the QRS complex, and the T wave compared to younger age groups, as previously reported in Thoroughbreds [30]. Another recent work also noted a tendency for an increase in QRS duration with age, as was observed in the present study [20]. In lead II, horses above 14 years of age showed a longer QT interval than other age groups. This longer QT interval may be related to increased heart mass [18], as evidence suggests that horses below 5 years of age are yet to complete their normal cardiac development [30]. Additionally, most of the older horses in this study had undergone an active athletic career over the years, which could have contributed to myocardial hypertrophy, although this hypothesis was not confirmed by echocardiography and thus remains speculative. However, in lead II, Group 1 (3 to 5 years) had a significantly longer RR interval than Group 3 (>14 years).

Regarding weight, no significant correlations were observed, likely due to the limited variation in weight among the horses. By the age of 3, Lusitano horses have typically reached their adult weight, resulting in minimal weight differences among the animals included in this study. 

The authors acknowledge that obtaining a more balanced number of males and females would have been ideal. However, the exclusion of pregnant females from the study resulted in a smaller number of females. Additionally, while the weight measurement was conducted using a scientifically reliable method, using a specialized balance for weighing horses could have provided greater sensitivity in the measurement. No testing with thoracic radiography, echocardiography, complete blood count, serum biochemical analyses, or urinalyses was conducted to rule out the presence of subclinical or underlying cardiac or pulmonary disease in the horses. However, prior to the ECGs, all horses underwent thorough physical examinations and were determined to be clinically normal. Finally, a larger sample size could have allowed the calculation of reference values for all electrocardiographic parameters.

## 5. Conclusions

This study presented electrocardiographic reference values for Lusitano horses, which can be used as important data for future studies. This study found that some of the values for Lusitano horses differ from those of other breeds, underscoring the importance of obtaining breed-specific electrocardiographic values. To our knowledge, this is the first description of healthy Lusitano horse electrocardiographic parameters in the equine veterinary literature.

## Figures and Tables

**Table 1:** Lusitano horse ECG reference values for the base–apex lead. Parameters for which it was not possible to calculate a reference value are indicated with a dash symbol “-”. IQR, interquartile range; RI, reference interval. Duration in seconds (s); amplitude in millivolts (mV).

ECG Characteristics			Median	IQR	RI	Lower Limit	Upper Limit
P wave	Simple P wave Duration		0.13	0.02	0.09–0.17	0.08–0.09	0.16–0.18
	Simple P wave Amplitude		0.27	0.06	-	-	-
	Bifid P wave	P1 Amplitude	0.12	0.05	0.08–0.26	0.07–0.09	0.20–0.43
		P2 Amplitude	0.31	0.12	0.18–0.51	0.17–0.18	0.45–0.55
	Biphasic P wave	P+	0.27	0.06	-	-	-
		P−	0.08	0.00	-	-	-
QRS	Q wave Amplitude		0.13	0.00	-	-	-
	R wave Amplitude		0.30	0.29	0.09–0.97	0.09–0.12	0.84–0.98
	S wave Amplitude		1.79	0.44	0.89–2.47	0.35–1.20	2.38–2.50
	QRS complex Duration		0.12	0.04	0.07–0.17	0.07–0.08	0.16–0.20
T wave	Biphasic T wave	T+ Amplitude	0.22	0.12	0.10–0.87	0.10–0.12	0.49–0.88
		T− Amplitude	0.59	0.51	0.12–1.94	0.10–0.24	1.43–2.15
	Negative T wave Amplitude		0.93	0.25	-	-	-
	Positive T wave Amplitude		0.56	0.28	-	-	-
	Simple T wave Duration		0.18	0.05	0.08–0.32	0.07–0.12	0.27–0.44
Intervals	PR interval		0.30	0.05	0.21–0.41	0.15–0.22	0.38–0.44
	QT interval		0.51	0.05	0.33–0.67	0.32–0.39	0.59–0.72
	RR interval		1.78	0.54	1.09–2.29	1.07–1.27	2.24–2.34

**Table 2:** Lusitano horse ECG reference values for the lead II. Parameters for which it was not possible to calculate a reference value are indicated with a dash symbol “-”. IQR, interquartile range; RI, reference interval. Duration in seconds (s); amplitude in millivolts (mV).

ECG Characteristics			Median	IQR	RI	Lower Limit	Upper Limit
P wave	Simple P wave Duration		0.11	0.02	0.06–0.15	0.05–0.07	0.14–0.15
	Simple P wave Amplitude		0.22	0.05	-	-	-
	Bifid P wave	P1 Amplitude	0.10	0.05	0.05–0.20	0.05–0.07	0.15–0.20
		P2 Amplitude	0.22	0.05	0.13–0.42	0.13–0.15	0.30–0.47
	Biphasic P wave	P+	0.22	0.05	-	-	-
		P−	-	-	-	-	-
QRS	Q wave Amplitude		0.13	0.15	0.04–0.43	0.03–0.06	0.34–0.43
	R wave Amplitude		0.45	0.44	0.08–1.43	0.07–0.12	0.18–1.53
	S wave Amplitude		0.20	0.33	-	-	-
	QRS complex Duration		0.08	0.04	0.04–0.16	0.04–0.05	0.13–0.17
T wave	Biphasic T wave	T+ Amplitude	0.17	0.14	0.07–0.68	0.05–0.09	0.40–0.87
		T− Amplitude	0.33	0.25	0.07–0.81	0.07–0.10	0.63–0.90
	Negative T wave Amplitude		0.37	0.14	-	-	-
	Positive T wave Amplitude		0.30	0.18	-	-	-
	Simple T wave Duration		0.17	0.06	0.08–0.31	0.04–0.08	0.28–0.33
Intervals	PR interval		0.28	0.06	0.21–0.38	0.16–0.21	0.34–0.39
	QT interval		0.51	0.07	0.41–0.63	0.41–0.42	0.59–0.71
	RR interval		1.70	0.47	1.09–2.31	0.92–1.17	2.24–2.51

**Table 3:** Electrocardiographic values in lead II and base–apex lead (median (IQR)) for each gender. ^a,b,c,d,e^ Superscript letter shows significant differences among genders. NP, not present; NA, not applicable. Duration in seconds (s); amplitude in millivolts (mV).

Variable			Lead II				Base–Apex Lead			
			Male (*n* = 63)		Female (*n* = 19)		Male (*n* = 63)		Female (*n* = 19)	
			Amplitude	Duration	Amplitude	Duration	Amplitude	Duration	Amplitude	Duration
P wave	Bifid P	P1 wave	0.10 (0.05)	0.12 (0.01)	0.10 (0.02)	0.10 (0.04)	0.12 (0.08)	0.12 (0.02)	0.10 (0.05)	0.13 (0.01)
		P2 wave	0.22 (0.05) ^a^		0.20 (0.05) ^a^		0.33 (0.10)		0.27 (0.12)	
	Simple P wave (P+)		0.20 (0.05)		0.25 (0.06)		0.27 (0.07)		NP	
	Biphasic P	P−	NP		NP		0.08 (0.00)		NP	
		P+	NP		NP		0.26 (0.06)		NP	
QRS	Q wave		0.12 (0.07)	0.08 (0.05)	0.25 (0.13)	0.08 (0.06)	0.13 (0.00)	0.13 (0.03) ^c^	NP	0.14 (0.05) ^c^
	R wave		0.51 (0.43) ^b^		0.22 (0.14) ^b^		0.32 (0.38)		0.26 (0.07)	
	S wave		0.20 (0.35)		0.19 (0.09)		1.79 (0.46)		1.78 (0.48)	
T wave	Biphasic T	T−	0.26 (0.25)	0.17 (0.07)	0.63 (0.61)	0.17 (0.03)	0.71 (0.41)	0.17 (0.05)	0.53 (0.15)	0.17 (0.05)
		T+	0.17 (0.13)		0.23 (0.12)		0.27 (0.16)		0.18 (0.11)	
	Negative T wave		0.30 (0.05)		1.00 (0.22)		1.02 (0.18)		0.53 (0.29)	
	Positive T wave		0.36 (0.15)		0.56 (0.26)		0.67 (0.28)		0.49 (0.31)	
Intervals	PR interval		NA	0.28 (0.05)	NA	0.26 (0.05)	NA	0.30 (0.05)	NA	0.33 (0.05)
	QT interval		NA	0.51 (0.08)	NA	0.51 (0.04)	NA	0.50 (0.05)	NA	0.53 (0.04)
	RR interval		NA	1.71 (0.40) ^d^	NA	1.55 (0.85) ^d^	NA	1.78 (0.51) ^e^	NA	1.66 (0.58) ^e^

**Table 4:** Electrocardiographic values in lead II and base–apex lead (median (IQR)) for each age group: Group 1, 3–5 years; Group 2, 6–14 years; Group 3, >14 years. ^a,b,c,d^ Superscript letter shows significant differences among age groups. NP, not present; NA, not applicable. Duration in seconds (s); amplitude in millivolts (mV).

Variable			Lead II						Base–Apex Lead					
			Group 1 (*n* = 22)		Group 2 (*n* = 43)		Group 3 (*n* = 17)		Group 1 (*n* = 22)		Group 2 (*n* = 43)		Group 3 (*n* = 17)	
			Amplitude	Duration	Amplitude	Duration	Amplitude	Duration	Amplitude	Duration	Amplitude	Duration	Amplitude	Duration
P wave	Bifid P	P1 wave	0.10 (0.03)	0.11 (0.04)	0.10 (0.05)	0.12 (0.02)	0.10 (0.05)	0.11 (0.04)	0.12 (0.07)	0.12 (0.02)	0.12 (0.05)	0.13 (0.02)	0.10 (0.05)	0.13 (0.02)
		P2 wave	0.20 (0.06)		0.23 (0.06)		0.20 (0.02)		0.32 (0.07)		0.33 (0.13)		0.30 (0.09)	
	Simple P wave (P+)		0.20 (0.01)		0.20 (0.09)		0.25 (0.03)		0.27 (0.13)		0.27 (0.05)		0.30 (0.05)	
	Biphasic P	P−	NP		NP		NP		0.08 (0.00)		NP		NP	
		P+	NP		NP		NP		0.26 (0.06)		NP		NP	
QRS	Q wave		0.14 (0.11)	0.07 (0.02)	0.10 (0.08)	0.09 (0.04)	0.25 (0.12)	0.08 (0.03)	NP	0.11 (0.04) ^a^	NP	0.12 (0.02) ^a^	0.13 (0.00)	0.14 (0.04) ^a^
	R wave		0.47 (0.28)		0.47 (0.59)		0.35 (0.30)		0.29 (0.16)		0.31 (0.28)		0.27 (0.37)	
	S wave		0.16 (0.32)		0.20 (0.35)		0.19 (0.15)		1.78 (0.30)		1.83 (0.53)		1.76 (0.62)	
T wave	Biphasic T	T−	0.27 (0.28)	0.16 (0.05)	0.30 (0.26)	0.16 (0.06)	0.38 (0.20)	0.17 (0.04)	0.67 (0.31)	0.18 (0.05) ^b^	0.50 (0.67)	0.18 (0.05) ^b^	0.59 (0.29)	0.21 (0.04) ^b^
		T+	0.20 (0.13)		0.17 (0.12)		0.15 (0.09)		0.25 (0.13)		0.22 (0.14)		0.18 (0.04)	
	Negative T wave		0.20 (0.13)		0.42 (0.11)		0.43 (0.03)		0.81 (0.00)		1.00 (0.16)		0.80 (0.61)	
	Positive T wave		0.15 (0.00)		0.30 (0.20)		0.35 (0.00)		0.48 (0.00)		0.86 (0.23)		0.48 (0.31)	
Intervals	PR interval		NA	0.27 (0.07)	NA	0.28 (0.06)	NA	0.28 (0.06)	NA	0.30 (0.06)	NA	0.29 (0.05)	NA	0.33 (0.03)
	QT interval		NA	0.49 (0.04) ^c^	NA	0.51 (0.05) ^c^	NA	0.55 (0.06) ^c^	NA	0.50 (0.05)	NA	0.50 (0.06)	NA	0.53 (0.10)
	RR interval		NA	1.73 (0.45) ^d^	NA	1.69 (0.43) ^d^	NA	1.58 (0.80) ^d^	NA	1.82 (0.43)	NA	1.70 (0.55)	NA	1.82 (0.46)

## Data Availability

No new data were created or analyzed in this study. Data sharing is not applicable to this article.

## References

[1] Fregin G. (1985). Electrocardiography. Vet. Clin. N. Am..

[2] Mitchell K. (2019). Equine Electrocardiography. Vet. Clin. N. Am. Equine Pract..

[3] Van Loon G. (2019). Cardiac Arrhythmias in Horses. Vet. Clin. N. Am. Equine Pract..

[4] Schwarzwald C. (2018). Disorders of the Cardiovascular System. Equine Internal Medicine: Fourth Edition.

[5] Barbesgaard L., Buhl R., Meldgaard C. (2010). Prevalence of Exercise-Associated Arrhythmias in Normal Performing Dressage Horses. Equine Vet. J..

[6] Leroux A., Detilleux J., Sandersen C., Borde L., Houben R., Haidar A., Art T., Amory H. (2013). Prevalence and Risk Factors for Cardiac Diseases in a Hospital-Based Population of 3434 Horses (1994–2011). J. Vet. Intern. Med..

[7] Hesselkilde E., Almind M., Petersen J., Flethøj M., Præstegaard K., Buhl R. (2014). Cardiac Arrhythmias and Electrolyte Disturbances in Colic Horses. Acta Vet. Scand..

[8] Morgan R., Raftery A., Cripps P., Senior J., McGowan C. (2011). The Prevalence and Nature of Cardiac Arrhythmias in Horses Following General Anaesthesia and Surgery. Acta Vet. Scand..

[9] Van Loon G., Patteson M. (2010). Electrophysiology and Arrhythmogenesis. Cardiology of the Horse.

[10] Da Silva Faria R., Vicente A., dos Santos R., Maiorano A., Curi R., Chardulo L., Vasconcelos Silva J. (2018). Genetic Diversity of Lusitano Horse in Brazil Using Pedigree Information. J. Equine Vet. Sci..

[11] Vicente A., Carolino N., Gama L. (2012). Genetic Diversity in the Lusitano Horse Breed Assessed by Pedigree Analysis. Livest. Sci..

[12] Guedes dos Santos R. (2008). Caracterizacón Genética de la Aptitud Deportiva Del Caballo Pura Sangre Lusitano a Partir de Variables Biocinemáticas al Trote.

[13] WBFSH World Breeding Federation for Sport Horses Studbook Rankings. http://www.wbfsh.org/GB.aspx.

[14] Bartolomé E., Milho S., Prazeres J. (2019). Genealogical and Morphological Analysis of Lusitano Purebred Horses Participating at International Dressage Competitions. Res. Vet. Sci..

[15] APSL Portuguese Lusitano Horse Breeders Association. https://www.cavalo-lusitano.com/.

[16] Bright J., Marr C. (2010). Introduction to Cardiac Anatomy and Physiology. Cardiology of the Horse.

[17] Ayala I., Montes A., Bernal L., Sandoval J., Gutierrez C. (1995). Electrocardiographic Values in Spanish-Bred Horses of Different Ages. Aust. Vet. J..

[18] Tavanaei-Manesh H., Dalir-Naghadeh B. (2010). Electrocardiographic Parameters in Purebred Kurd Horse. J. Anim. Vet. Adv..

[19] Ayala I., Gutierrez C., Benedito J., Prieto F., Montes A., Ayala I. (2000). Morphology and Amplitude Values of the Electrocardiogram of Spanish-Bred Horses of Different Ages in the Dubois Leads System. Vet. Res..

[20] Diniz M., Muzzi R., Muzzi L., Alves G. (2008). Eletrocardiographic Study in Horses of the Mangalarga Marchador Breed. Arq. Bras. Med. Vet. Zootec..

[21] Fregin G. (1982). The Equine Electrocardiogram with Standardized Body and Limb Positions. Cornell. Vet..

[22] Ayala I., Gutierrez C., Benedito J., Hernandez J., Castillo C., Lopez M., Alonso L., Miranda M., Montes A. (1999). Morphology and Amplitude Values of the P and T Waves in the Electrocardiograms of Spanish-Bred Horses of Different Ages. J. Vet. Med. A.

[23] Wagner E., Tyler P. (2011). A Comparison of Weight Estimation Methods in Adult Horses. J. Equine Vet. Sci..

[24] Silvestre-Ferreira A., Cotovio M., Maia M., Queiroga F., Pires M., Colaço A. (2018). Reference Intervals for Haematological Parameters in the Lusitano Horse Breed. Acta Vet. Hung..

[25] Pimenta J., Pires I., Prada J., Cotovio M. (2023). E-Cadherin Immunostaining in Equine Melanocytic Tumors. Animals.

[26] Escudero A., González J., Benedito J., Prieto F., Ayala I. (2009). Electrocardiographic Parameters in the Clinically Healthy Zamorano-Leones Donkey. Res. Vet. Sci..

[27] Geffré A., Concordet D., Braun J., Trumel C. (2011). Reference Value Advisor: A New Freeware Set of Macroinstructions to Calculate Reference Intervals with Microsoft Excel. Vet. Clin. Pathol..

[28] Evans D., Young L. (2010). Cardiac Responses to Exercise and Training. Cardiology of the Horse.

[29] Ramos S., Pinto A., Cardoso M., Alexandre N., Bettencourt E., Monteiro S., Gama L.T. (2020). Prevalence of Radiographic Signs of Osteoarthritis in Lusitano Purebred Horses. J. Equine Vet. Sci..

[30] Fernandes W., Larsson M., Alves A., Fantoni D., Belli C. (2004). Electrocardiographic Parameters in Clinically Healthy Thoroughbred Horses. Arq. Bras. Med. Vet. Zootec..

[31] Kenchaiwong W., Sangpo P., Kusol A., Pontaema T., Lerdweeraphon W. (2022). The Position of Ground Electrode Affects Electrocardiographic Parameters in Horses. Vet. World.

[32] Costa G., Illera M., García A. (1985). Electrocardiographical Values in Non-Trained Horses. Zentralblatt Vet. A.

[33] Melchert A., Laposy C., Guasi V., Valle H., Santos G. (2012). Eletrocardiografia Computadorizada Em Cavalos Puro-Sangue Lusitano Submetidos a Exercício Físico. Arq Bras. Vet. Zootc.

[34] Nasser A., Mohammad R., Mohammad G., Ali R., Iradj N. (2002). The ECG of the Turkman Horse Using the Standard Lead Base Apex. J. Equine Vet. Sci..

[35] Ayala I., Montes A., Fernández del Palacio M., Panizo C. (1994). Studies on the Electrocardiogram of the Horse. An. De Vet. De Murcia.

[36] Hendricks B., Hendricks B.L. (2002). International Encyclopedia of Horse Breeds.

[37] Physick P., Colahan P., Mayhew I.G., Merritt A., Moore J. (1991). Diseases of the Cardiovascular System. Equine Medicine and Surgery.

[38] Jones W., Jones W. (1991). The Cardiovascular System. Equine Sports Medicine.

[39] Muylle E., Oyaert W. (1977). The Genesis of the Different Configurations of the “QRS” Complex. Zentralblatt Vet. A.

[40] Pampana S., Sgorbini M., Bizzete M. (2004). Niccolai Cesare ECG Evaluation in Trotter Horses Using Standard Bipolar Limb Leads and Standard Lead Base-Apex. Ippologia.

